# XBB.1, BQ1.1 and atypical BA.4.6/XBB.1 recombinants predominate current SARS-CoV-2 Wavelets with flu-like symptoms in Cameroon: A snapshot from genomic surveillance

**DOI:** 10.1371/journal.pgph.0003153

**Published:** 2024-05-10

**Authors:** Joseph Fokam, Ezechiel Ngoufack Jagni Semengue, Davy-Hyacinthe Gouissi Anguechia, Naomi-Karell Etame, Désiré Takou, Nadia Mandeng, Minelle Aurelie Kengni Ngueko, Grace Beloumou Angong, Sandrine Djupsa Ndjeyep, Collins Chenwi Ambe, Alex Durand Nka, Evariste Molimbou, Audrey Rachel Mundo Nayang, Larissa Gaëlle Moko Fotso, Derrick Tambe Ayuk Ngwese, Pamela Patricia Tueguem, Carlos Michel Tommo Tchouaket, Aude Christelle Ka’e, Nadine Fainguem, Cyrille Abega Abega, Edie-Gregory Halle-Ekane, Linda Esso, Alain Georges Etoundi Mballa, Judith Shang, Clement B. Ndongmo, Giulia Cappelli, Sofonias Kifle Tessema, Anne-Cecile Z-K Bissek, Vittorio Colizzi, Alexis Ndjolo, Carlo-Federico Perno, Nicaise Ndembi

**Affiliations:** 1 Chantal BIYA International Reference Centre for Research on HIV/AIDS Prevention and Management, Yaoundé, Cameroon; 2 Faculty of Health Sciences, University of Buea, Buea, Cameroon; 3 National Public Health Emergency Operations Centre, Ministry of Public Health, Yaoundé, Cameroon; 4 Central technical Group, National AIDS Control Committee, Ministry of Public Health, Yaoundé, Cameroon; 5 Faculty of Medicine and Biomedical Sciences, University of Yaoundé I, Yaoundé, Cameroon; 6 Department of Experimental Medicine, University of Rome “Tor Vergata”, Rome, Italy; 7 Faculty of Health Sciences, University of Bamenda, Bamenda, Cameroon; 8 Faculty of Science and Technology, Evangelic University of Cameroon, Bandjoun, Cameroon; 9 School of Health Sciences, Catholic University of Central Africa, Yaoundé, Cameroun; 10 Department of Disease, Epidemic and Pandemic Control, Ministry of Public Health, Yaoundé, Cameroon; 11 United States Centres for Diseases Control and Prevention, DGHT, Yaoundé, Cameroon; 12 National Research Council, Rome, Italy; 13 Africa Centres for Disease Control and Prevention (Africa CDC), Addis Ababa, Ethiopia; 14 Division of Health Operational Research, Ministry of Public Health, Yaounde, Cameroon; 15 Bambino Gesu’ Children’s Research Hospital, Rome, Italy; Bennett University, INDIA

## Abstract

As of December 2022, Cameroon had observed a slight resurgence of COVID-19, raising concerns on genomic surveillance of related-SARS-CoV-2 variants under circulation. Following a laboratory-based survey, positive SARS-CoV-2 samples detected from December-2022 through March-2023 were processed for targeted sequencing at the Chantal BIYA International Reference Centre (CIRCB) in Yaoundé-Cameroon. From all positive cases detected, 13 were successfully sequenced (mean age 34 years, 70% female); the majority of the cases were unvaccinated (70%, 9/13) and symptomatic (92%, 12/13); all with flu-like symptoms (100%, 12/12). Following RT-PCR, the median cycle threshold was 22.23 [18–24] for the N gene; and 24.09 [20–26] for the ORF gene, underscoring high viral loads. Phylogenetic analysis of nucleotide sequences identified four major sub-variants in circulation, of which BA.5 (3/13), the recombinants BQ.1.1 (4/13), XBB.1 (4/13) and novel atypical variant of BA.4.6/XBB.1 (2/13). This snapshot surveillance indicates the introduction/emergence and circulation of new Omicron sub-variants, all accompanied by minor/mild symptoms. However, these new sub-variants and recombinants call for continuous genomic surveillance to prevent further resurgence of Covid-19 epidemiological wave.

## 1. Introduction

Since the start of COVID-19 pandemic, Cameroon has experienced up to 5 major waves [1–3]; with the first being reported from March to September 2020 and driven by the original Wuhan strain (100%); the second wave was reported from October 2020 to May 2021 and driven mainly by alpha and beta variants (90%); the third wave from June to October 2021 and driven mainly by delta variants (90%); the fourth wave from December 2021 to March 2022 and driven by the Omicron (B.1.1.529) variant (100%); while the fifth wave was reported from July 2022 to March 2023, with mild symptoms but without delineating the circulating viral strains [2,3]. Of note, the Omicron (B.1.1.529) is a variant of SARS-CoV-2 reported to the World Health Organization (WHO) by the Network for Genomics Surveillance in South Africa on 24 November 2021 [4–6]. It was first detected in Botswana and rapidly became the predominant variant in circulation worldwide, with different sub-variants that have emerged from the original omicron lineage [4,7,8].

Since December 2022, Cameroon has observed a slight resurgence of cases with flu-like symptoms, raising concern about the potential circulation of new SARS-CoV-2 variants of concern. It would therefore be of great relevance to ensure ongoing genomic surveillance to inform public health response on possible impact of circulating variants on disease severity and preventive measures at population level. In this present investigation, Cameroonian patients positive for COVID-19 with flu-like symptoms were enrolled within the frame of genomic surveillance supported by the EDCTP PERFECT-Study. Of note, the EDCTP-PERFECT study aimed at evaluating the diagnostic performance of SARS-CoV-2 assays on well-characterized COVID-19 cases, including variant detection (https://edctp-perfect-study.com/).

## 2. Methods

### 2.1. Study type and setting

A laboratory-based survey was conducted on COVID-19 PCR positive nasopharyngeal samples. Testing and sequencing were conducted at the virology Laboratory of the “Chantal BIYA” International Reference Centre in Yaoundé, Cameroon.

### 2.2. Processing of the samples

RNA was extracted from 200 μL nasopharyngeal clinical swabs samples using the DaAn gene viral RNA Mini kit according to the manufacturer’s protocol. SARS-CoV-2 positivity was confirmed by real-time PCR using the DaAn gene rRT-PCR assay. Targeted sequencing of the spike protein was then initiated on viral RNA extracts following genotyping specific for SARS-CoV-2, adapted from published in-house viral genotyping protocols.

### 2.3. Confirmation of SARS-CoV-2 positivity

RT-PCR was conducted using the DaAn gene assay as per manufacturer’s instructions (Guangzhou, Guangdong Province, China). RT-PCR amplification was performed using QuantStudio qPCR Systems (Scientific Thermofisher). The protocol used probes targeting the open reading frame (ORF) gene and the nucleocapsid (N) protein gene, with a lower limit of detection of 500 copies/mL and an amplification reaction of 45 cycles.

### 2.4. Amplification and sequencing of SARS-CoV-2

The protocol for SARS-CoV-2 amplification and sequencing is detailed elsewhere [9]. Briefly, viral RNA was reverse-transcribed and amplified using the kit One-Step Invitrogen (SuperScript One-Step for long templates RT-PCR; Foster City, CA) and 2 different primers (5’-3’); **38F** (-GTC AGT GTG TTA ATC TTA CAA CCA G-**)** as the forward, and **1191R** (-TGC ATA GAC ATT AGT AAA GCA GAG A-**)** as the reverse, (the given position refers to the Wuhan strain of SARS-CoV-2). For each PCR reaction, positive and negative controls were used to ensure the effectiveness of the reaction and the absence of cross-contamination, respectively. Amplification results were revealed after agarose-gel electrophoresis and positive results were kept for the sequencing process. PCR products were then purified through the ExoSAP-IT kit (Applied Biosystems, Lithuania). Sequencing was performed with four different overlapping primers: **38F** (-GTC AGT GTG TTA ATC TTA CAA CCA G-**), 514F** (-TCT CAG CCT TTT CTT ATG GAC CT-**), 655R** (-CCT GAG GGA GAT CAC GCA CTA-**) and 1191R** (-TGC ATA GAC ATT AGT AAA GCA GAG A-**)**. The sequencing product was purified by gel filtration chromatography using Sephadex G-50 resin (Sigma-Aldrich) to eliminate excess primers, unincorporated dideoxynucleotides (ddNTPs) and salts. Capillary electrophoresis was performed on Applied Biosystems 3500 genetic analyzer (Applied Biosystems, Tokyo, Japan).

### 2.5. SARS-CoV-2 sequence analysis

Sequences were aligned, assembled and edited by the reference sequence using Seqscape V2.7. Spike sequences were interpreted using the COV19 Stanford algorithm (https://covdb.stanford.edu). All the sequences obtain were blasted and analyzed on NCBI covid database (https://www.ncbi.nlm.nih.gov/activ) and subvariants were confirmed following molecular phylogeny by using MEGA 11 with reference sequences downloaded on the GISAID database (https://gisaid.org) through Audacity instant app.

### 2.6. Ethical considerations

The present research was approved by the Cameroon national committee for human health research (N°2020/05/1227/CNERSH/SP); all participants gave their written consent at inclusion and the CIRCB General Directorate issued administrative authorization for the study. For purpose of confidentiality and privacy, data were collected and processed using unique identifiers, and secured in a password encrypted database, with restricted access only to key investigators.

## 3. Results

**Fig 1:**
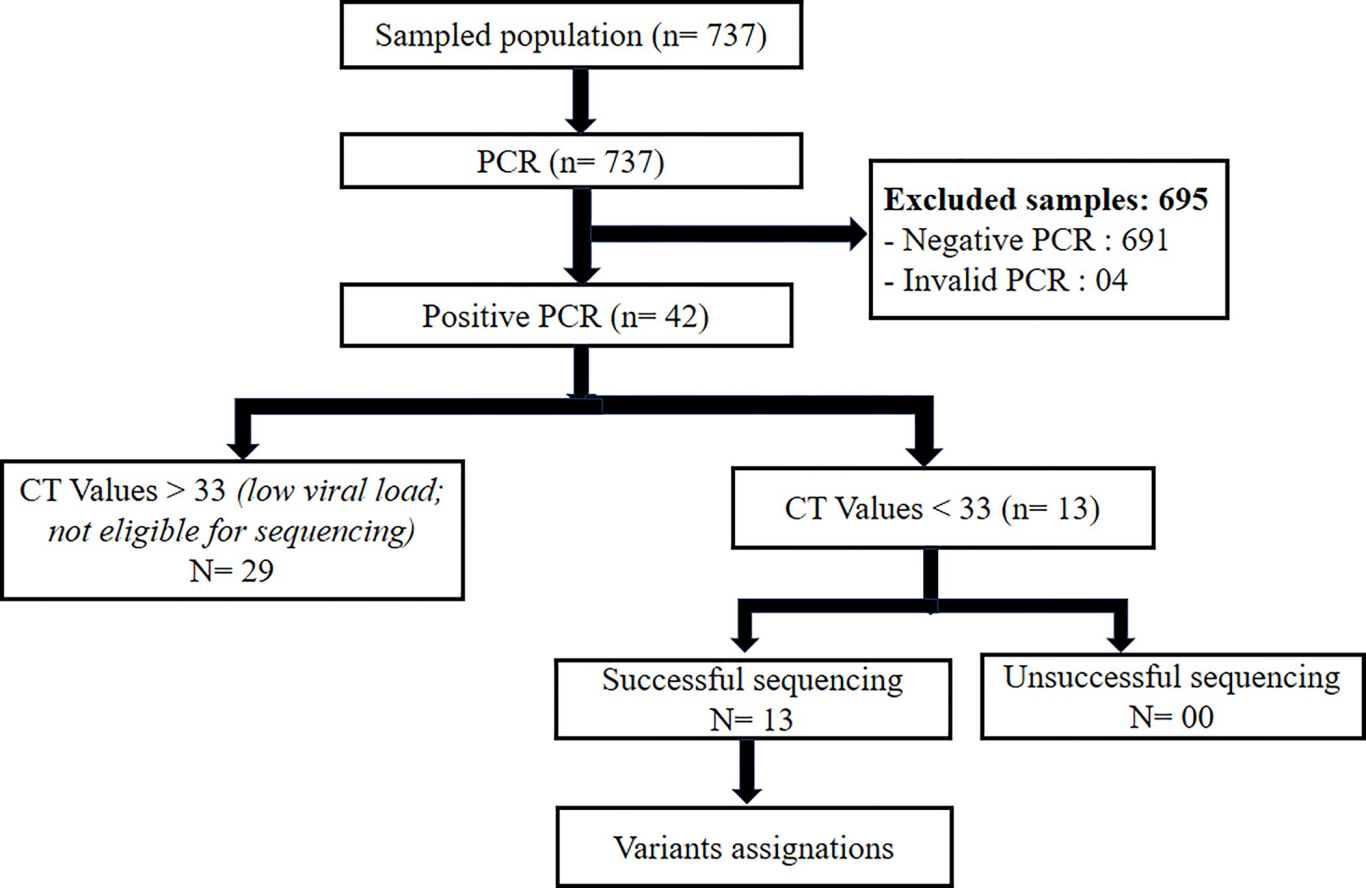
Flowchart of the sampling procedure.

Our sample population was drawn from a total of 737 participants tested for SARS-CoV-2 real-time qPCR. Of these, 42 (5.7%) qPCR positive for SARS-CoV-2. Among the positive cases (n = 42), 13 samples were eligible for sequencing based on CT values (i.e. with a CT < 33 amplification cycles) (Fig 1).

### 3. 1. Sociodemographic and clinical features

The 13 cases included were all Cameroonians, mainly female (70%) with a mean age of 34 ±7 years. The majority of these cases 92.31% (12/13) presented mild symptoms ranging from headaches (58.33%; 7/12), fever (50%; 6/12), asthenia (41.67%; 5/12), cough (33.33%; 4/12), sore throat (33.33%; 4/12), shortness of breath (25%; 3/12), shivering (25%; 3/12), runny nose (16.67%; 2/12), nasal discharges (16.67%; 2/12), diarrhea (8.33%; 1/12), loss of taste (8.33%; 1/12), arthralgia (8.33%; 1/12) and myalgia (8.33%; 1/12). About 70% (9/13) were non-vaccinated. Following RT-PCR, the majority of cases were found with higher viral loads; median Ct was 22.23 [IQR: 18–24] for the N gene; and 24.09 [IQR: 20–26] for the ORF gene. Detailed description of individual profile and mutations detected by viral strain, is provided in Table 1.

**Table 1:** Details of omicron sub-variants detected at the CIRCB over the period December 2022 to March 2023.

N°	Patient	Sampling collection date	Age and gender	Symptomatology and Vaccination Status	Mutations on the S protein of SARS-CoV-2	Omicron Sub-variants	Viral load (cycle threshold)
01	Patient 1	22/12/2022	32 years Female	- Not vaccinated -Asymptomatic	Δ69–70, G142D, Q183QE, V213G, G339D, L368X Variant : OMICRON	Sub-variant : BA.5	ORF Gene = 18.00; N Gene = 16.30;
02	Patient 2	23/12/2022	29 years Male	- Not vaccinated - Symptomatic (Fever, asthenia, runny nose, headache,)	Δ69–70, G142D, Δ144, V213G, G339D, R346T Variant : OMICRON	Sub-variant : BA.5	ORF Gene = 15.05; N Gene = 12.91;
03	Patient 3	09/02/2023	27 years Female	- Not vaccinated - Symptomatic (Fever, sore throat, headache, diarrhea)	R21L, Q23T, L24R, P25T, P26Q, A27S, D80H, V83A, G142D, Δ144, H146Q, Q183E, V213E, G252V, G339H, R346T, L368I, S371F, S373P, S375F, T376A Variant : OMICRON	Sub-variant : XBB.1.1	ORF Gene = 24.00; N Gene = 26.00
04	Patient 4	14/02/2023	40 years Female	- Vaccinated - Symptomatic (Fever, nasal discharge, muscle pain, asthenia)	A27X, V83A, G142D, Δ144, H146Q, Q183E, V213E, G252V, G339H, R346T, L368I, S371F, S373P, S375F, T376A Variant : OMICRON	Sub-variant : BA.4.6/XBB.1	ORF Gene = 20.00; N Gene = 18.00
05	Patient 5	01/03/2023	52 years Male	- Vaccinated - Symptomatic (Cough, nasal discharge, headache, asthenia)	Δ69–70, G142D, K147E, F186Y, Δ187–188, V213G, G339D, R346T Variant : OMICRON	Sub-variant : BQ.1.1	ORF Gene = 24.93; N Gene = 20.18
06	Patient 6	27/02/2023	33 years Female	- Not vaccinated - Symptomatic (Sore throat)	Δ69–70, G142D, K147E, F186Y, Δ187–188, V213G, G339D, R346T Variant: OMICRON	Sub-variant : BQ.1.1	ORF Gene = 21.00; N Gene = 20.00
07	Patient 7	01/03/2023	41 years Female	- Not vaccinated - Symptomatic (Fever, shivering, sore throat, headache)	V83A, G142D, Δ144, H146Q, Q183E, V213E, G252V, G339H, R346T Variant: OMICRON	Sub-variant : XBB.1.1	ORF Gene = 26; N Gene = 24
08	Patient 8	02/03/2023	31 years Female	- Vaccinated - Symptomatic (Sore throat, headache)	Δ69–70, G142D, K147E, F186Y, Δ187–188, V213G, G339D, R346T Variant : OMICRON	Sub-variant : BQ.1.1	ORF Gene = 26.00; N Gene = 24.00
09	Patient 9	02/03/2023	28 years Female	- Vaccinated - Symptomatic (Shortness of breath, shivering)	Δ69–70, G142D, K147E, F186Y, Δ187–188, V213G, G339D, R346T Variant : OMICRON	Sub-variant : BA.5	ORF Gene = 33.00; N Gene = 31.00
10	Patient 10	07/03/2023	40 years Male	- Not vaccinated - Symptomatic (Shortness of breath, shivering, joint pain, headaches, asthenia)	V83A, G142D, Δ144, H146Q, Q183E, V213E, G252V, G339H, R346T, L368I, S371F, S373P, S375F, T376X Variant: OMICRON	Sub-variant : BA.4.6/XBB.1	ORF Gene = 17.50; N Gene = 15.60
11	Patient 11	06/03/2023)	28 years Female	- Not vaccinated - Symptomatic (Cough, shortness of breath, headache, asthenia)	Y28X, V42X, Δ69–70, G142D, K147E, F186Y, Δ187–188, V213G, G339D, R346T, C361X Variant : OMICRON	Sub-variant : BQ.1.1	ORF Gene = 33.00; N Gene = 28.50
12	Patient 12	14/03/2023	27 years Female	- Not vaccinated - Symptomatic (Fever, cough, runny nose, headache, loss of taste)	R21L, Q23T, L24R, P25T, P26Q, A27S, D80H, V83A, G142D, Δ144, H146Q, Q183E, V213E, G252V, G339H, R346T Variant: OMICRON	Sub-variant : XBB.1	ORF Gene = 24.09; N Gene = 22.23
13	Patient 13	15/03/2023	40 years Male	- Not vaccinated - Symptomatic (Fever, cough,)	D80H, V83A, G142D, Δ144, H146Q, Q183E, V213E, G252V, G339H, R346T Variant: OMICRON	Sub-variant : XBB.1	ORF Gene = 25.00; N Gene = 23.00

### 3. 2. Subvariants’ distribution

**Fig 2:**
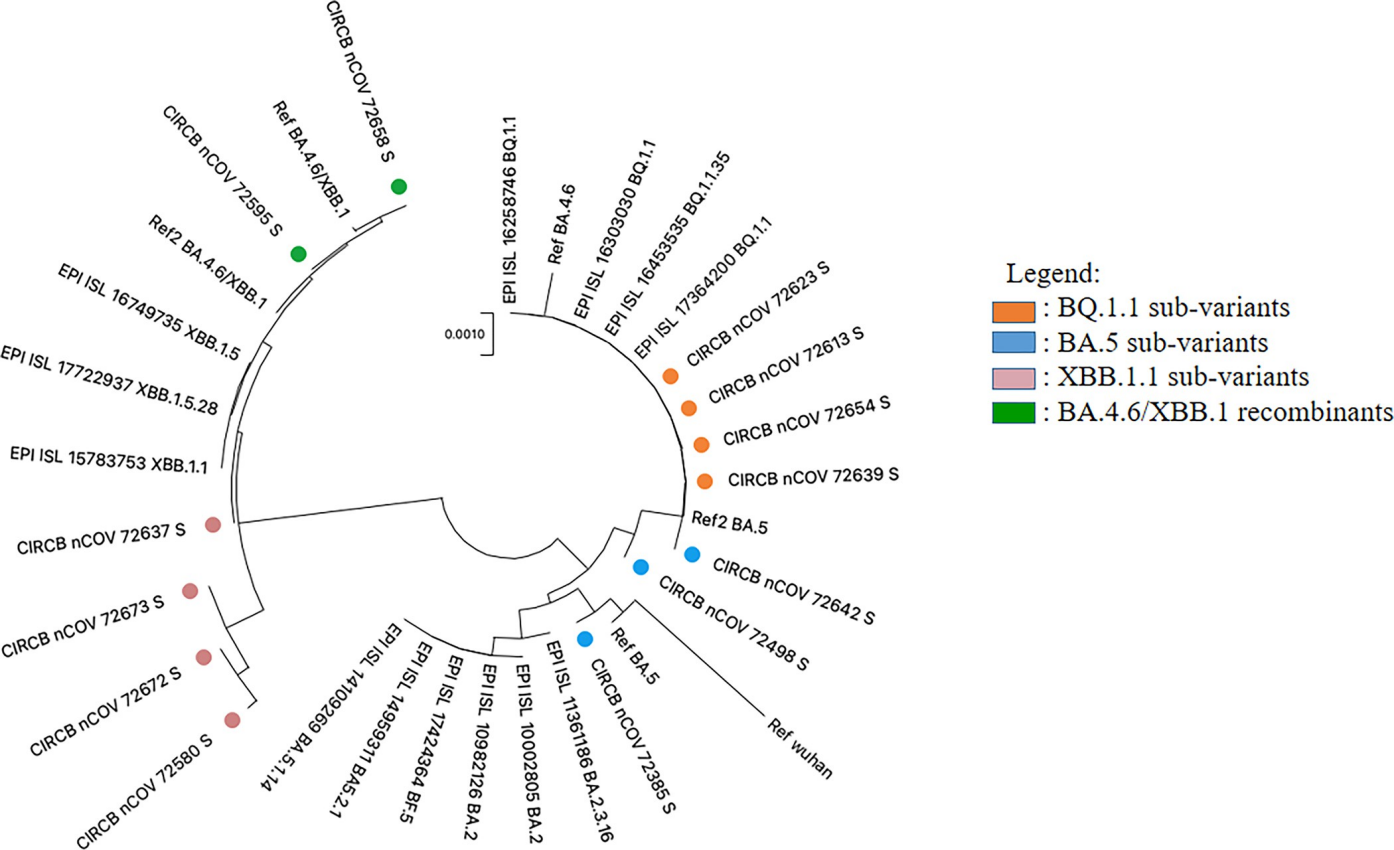
Phylogenetic tree of SARS-CoV-2 sequences obtained.

All cases were unsurprisingly Omicron variants (100%) following molecular phylogeny, of which three sub-variants BA.5 (3/13), four recombinants of BQ.1.1 (4/13) and of XBB.1 (4/13), but also two cases of atypical novel recombinants of sub-variants (BA.4.6/XBB.1 (2/13); as depicted in Fig 2.

## 4. Discussion

Our primary aim was to provide genomic signatures to substantiate the current epidemiological trend of SARS-CoV-2 infection in Cameroon, with evidence on variants that were dominant in the observed wavelet. The few numbers of cases herein presented underscores the low prevalence of SARS-CoV-2 during the study period [1,3]. This clearly reflects the epidemiological situation during low-positivity rates of an outbreak such as COVID-19, as positive cases during this period often have high CT-values (i.e. low viral load). Additionally, low viral circulation likely results from efforts to achieve herd immunity through vaccination and natural infection in sub-Saharan Africa [1,3].

All cases were screened between December 2022 and March 2023, which are in line with the global circulation of omicron sub-variants [5,7,8,10]. This in turn justifies the low severity (mild symptoms), as the omicron wave was characterized by fewer patients admitted to hospital, less severe illness, and a lower case-fatality rate [4,5,7,8,10–14]. The mild symptoms observed despite higher viral loads and low vaccination coverage further support of the higher risk of viral transmissibility and low severity in a context of population immunity by viral exposure during previous waves [15]. The mild symptoms, observed among immune-competent individuals, might be concerning among vulnerable populations [11–14]. Interestingly, the most common mutations found in this study were G142D (100%), G339DH (100%) and the R346T (92.3%). Following interpretation from the Stanford COVID-19 database, G142D is a common N-terminal domain (NTD) mutation present in the delta and omicron variants that interferes with the neutralization of many NTD-binding monoclonal antibodies (mAbs); as for G339D, it is a receptor biding domain (RBD) core mutation that is present in the omicron variant whereas G339H is present in BA.2.75 and XBB which does not appear to reduce mAb susceptibility. Finally, R346T was reported from BA.4.6 lineage but not in the ancestral BA4/5 variant; it is associated with reduction in susceptibility to Cilgavimab/COV2-2130/AZD1061 (a mAbs) in vitro database (https://covdb.stanford.edu).

From a molecular epidemiology perspective, sub-variants reported here were similar to the global trends. However, the two atypical recombinants (BA.4.6/XBB.1), confirmed by molecular phylogeny, indicate ongoing viral recombination (novel mutations) and risk of emergence of a new variant subsequently [6,7]. Even though this study lays emphasis on few cases, our findings also call for further characterization of these potential new recombinants in order to fully describe their mutational patterns. These insights underscore the need to reinforce routine genomic surveillance to detect potential resurgence timely, the impact of novel mutations on vaccine/immune-escape, to mitigate global outbreaks.

The emergence and circulation of new Omicron sub-variants and recombinants are driving the current trend of COVID-19 epidemiology in Cameroon with mid symptoms. However, the emergence of atypical recombinants (BA.4.6/XBB.1) calls for routine genomic surveillance to timely detect and track novel strains, related disease severity and risk of transmission for optimal pandemic control.
